# Is recurrence possible in coronavirus disease 2019 (COVID-19)? Case series and systematic review of literature

**DOI:** 10.1007/s10096-020-04057-6

**Published:** 2020-10-10

**Authors:** Anna Gidari, Marco Nofri, Luca Saccarelli, Sabrina Bastianelli, Samuele Sabbatini, Silvia Bozza, Barbara Camilloni, Igino Fusco-Moffa, Claudia Monari, Edoardo De Robertis, Antonella Mencacci, Daniela Francisci

**Affiliations:** 1 Department of Medicine, Clinic of Infectious Diseases, “Santa Maria della Misericordia” Hospital, University of Perugia, Perugia, Italy; 2 Department of Anesthesiology, Intensive Care and Pain therapy Center, “Santa Maria della Misericordia” Hospital, University of Perugia, Perugia, Italy; 3 Department of Medicine, Medical Microbiology Section, University of Perugia, Perugia, Italy; 4 Department of Prevention, Local Health Unit Umbria 1, Travel Medicine Unit, Perugia, Italy

**Keywords:** COVID-19, SARS-CoV-2, Recurrence, Reactivation, Re-infection

## Abstract

Can a patient diagnosed with severe acute respiratory syndrome coronavirus-2 (SARS-CoV-2) be infected again? This question is still unsolved. We tried to analyze local and literature cases with a positive respiratory swab after recovery. We collected data from symptomatic patients diagnosed with SARS-CoV-2 infection in the Italian Umbria Region that, after recovery, were again positive for SARS-CoV-2 in respiratory tract specimens. Samples were also assessed for infectivity in vitro. A systematic review of similar cases reported in the literature was performed. The study population was composed of 9 patients during a 4-month study period. Among the new positive samples, six were inoculated in Vero-E6 cells and showed no growth and negative molecular test in culture supernatants. All patients were positive for IgG against SARS-CoV-2 nucleoprotein and/or S protein. Conducting a review of the literature, 1350 similar cases have been found. The presumptive reactivation occurred in 34.5 days on average (standard deviation, SD, 18.7 days) after COVID-19 onset, when the 5.6% of patients presented fever and the 27.6% symptoms. The outcome was favorable in 96.7% of patients, while the 1.1% of them were still hospitalized at the time of data collection and the 2.1% died. Several hypotheses have been formulated to explain new positive respiratory samples after confirmed negativity. According to this study, the phenomenon seems to be due to the prolonged detection of SARS-CoV-2 RNA traces in respiratory samples of recovered patients. The failure of the virus to replicate in vitro suggests its inability to replicate in vivo.

## Introduction

Severe acute respiratory syndrome coronavirus-2 (SARS-CoV-2) is the etiologic agent of coronavirus disease 2019 (COVID-19) that has been declared a global pandemic by the World Health Organization (WHO) in March 2020. SARS-CoV-2 was discovered in December 2019, in Wuhan City (the capital of Hubei province), China [1].

The origin of the virus remains unknown. However, newly diagnosed cases were initially linked to the Huanan Seafood Wholesale Market where people can buy animals that are butchered in loco. The virus was identified as a novel enveloped RNA beta-coronavirus that has been named SARS-CoV-2 [2].

As of September 2020, the total worldwide confirmed cases are 30,949,804 [3].

Albeit our knowledge of this virus has improved, it is still a challenging matter whether patients with SARS-CoV-2 infection will reactivate the illness, and which risk factors predict eventual recurrence. Unraveling this topic is important to better understand the immune response to the virus and contain disease transmission.

After a prolonged scientific debate, the characteristics of a patient that can be discharged from the hospital have been standardized, in particular, improvement of symptoms (normal temperature lasting longer than 3 days and no more respiratory symptoms) and two negative swabs collected at least 24 h apart [4]. However, from June 17, WHO simplified criteria for discharge: for symptomatic patients, 10 days after symptom onset, plus at least 3 additional days, without symptoms (including no fever and respiratory symptoms) is enough to discharge patients from isolation [4].

However, several authors observed that in some cases patients presented a new positive respiratory sample after discharge. It is not clear yet what this means in terms of clinical course and contagiousness.

Here, we report the clinical and virologic findings of nine patients with a positive swab that had the criteria for discharge, in the Italian Umbria Region, from March to September. We also systematically revised all the similar cases reported in the literature so far, evaluating the possibility that COVID-19 may recur after recovery.

## Materials and methods

### Patients, molecular tests, and in vitro viral infectivity

Criteria for patients’ selection were diagnosis of SARS-CoV-2 infection [5]; the subsequent meeting of criteria for hospital discharge (improvement of symptoms and two negative swabs collected at least 24 h apart) [4]; and a positive respiratory sample collected after discharge.

All subjects provided informed oral consent to clinical data collection. The study was approved by the Ethics Committee of the Umbria Region (protocol number 18344/20/OV). Respiratory samples (nasopharyngeal swabs, sputum, tracheal aspirate, or bronchoalveolar lavage, BAL, fluid) were tested for SARS-CoV-2 RNA by a commercial reverse transcriptase real-time PCR assay (RT-PCR assay, Allplex™ 2019-nCoV Assay, Seegene, Seoul) and/or with the Xpert® Xpress SARS-CoV-2 (Cepheid, Sunnyvale, CA, USA). In particular, Allplex™ 2019-nCoV Assay was performed according to the manufacturer’s instructions, using 300 μL of respiratory samples and 10 μL of the provided internal control (IC). The envelope (*E*) gene (specific of the subgenus Sarbecovirus), the nucleocapsid gene (*N*), and the RNA-dependent-RNA-polymerase (*RdRP)* genes (both specifics of the SARS-CoV-2) were the targeted genes of the RT-PCR. The assay was considered valid if the cycle threshold (Ct) value of the IC was ≤ 40. Samples with 2 or 3 viral targets (Ct ≤ 40) were considered positive. Samples with only 1 target (whatever Ct) or with 2 or 3 targeted genes > 40 Ct were considered indeterminate, requiring a new specimen for retesting. Samples were considered negative in the absence of any targeted gene. The fully automated Xpert Xpress® SARS-CoV-2 assay was performed on the GeneXpert® platform (Cepheid) according to the manufacturer’s instructions, loading 300 μL of the sample into a single-use disposable cartridge. Targets were E and N2 genes. After results were automatically passed, they were interpreted as positive if both E and N2 targets were detected. If a gene alone was detected, the sample was considered indeterminate, and a new sample was retested.

The virus isolation was conducted in a biosafety level-3 facility. Samples tested positive for the SARS-CoV-2 in real-time RT-PCR analyses were mixed with a 1:1 nystatin (10,000 U/mL) and penicillin-streptomycin (10,000 U/mL) mixture in a 1:4 ratio, and left to react at 4 °C for 1 h. The samples were then centrifuged at 400×g for 10 min, and the supernatant was used as the inoculant. For the cell inoculation, Vero E6 (ATCC® -1586) were maintained in Eagle’s minimum essential medium (MEM) supplemented with 10% fetal bovine serum, and 1% penicillin-streptomycin at 37 °C in the presence of 5% CO_2_. On the day prior to inoculation, the cells were seeded at 0.5 × 10^4^ cells/cm^2^ into a T25 flask. The inoculated cells were cultured for 5 days as above described. To evaluate the viral replication process, the cytopathic effect was observed at different exposure times, and RNA from the cell culture supernatant was extracted and assessed for the presence of SARS-CoV-2 using real-time RT-PCR [6, 7]. The titer of IgG against the SARS-CoV-2 S1/S2 subunit of spike protein was measured with CLIA LIAISON® SARS-CoV-2 S1/S2 IgG (DiaSorin).

### Systematic review

Following the Preferred Reporting Items for Systematic Reviews and Meta-Analyses (PRISMA) Statement protocol [8], a systematic review has been performed concerning the patients with a diagnosis of COVID-19 that, after clinical and virological recovery, presented a new positive respiratory sample (swab, sputum, saliva, tracheal aspirate, or BAL).

A systematic search on PubMed and Google Scholar was performed from January to September 1, 2020. We used the following searching strategy: every search included the terms “SARS-CoV-2” or “COVID-19” and “reactivation” or “recurrence” or “relapse.” Furthermore, we performed the following research: “(COVID-19 or SARS-CoV-2) and positive and (recovered or discharged).” Each article was analyzed to establish eligibility criteria and pertinency to our research. The inclusion criteria were as follows: English, Italian, or Spanish languages; pertinence on the question; articles that reported sufficient patients’ information such as age, sex, and clinical and radiological presentation; treatment; and outcome.

The exclusion criteria were as follows: different languages from those mentioned above and abstract and posters from conference proceedings since they did not go through peer review.

Data were summarized using descriptive analysis where possible. We included our presented cases in the analysis. Statistical analysis was performed with Prism GraphPad 8 software. The figure was created using Microsoft PowerPoint 2020.

## Results

### Study population

From March to September 1, 2020, 9 patients meeting the inclusion criteria were included in the study. Patient characteristics and laboratory findings are summarized in Table 1.

**Table 1:** Severity of symptoms, laboratory exams, serology for severe acute respiratory syndrome coronavirus 2 (SARS-CoV-2), and SARS-CoV-2 culture at the first admission (where available) and at the presumptive recurrence of coronavirus disease 2019 (COVID-19)

	Case 1		Case 2	Case 3	Case 4	Case 5	Case 6	Case 7	Case 8		Case 9
	Admission	New positive	New positive	New positive	New positive	New positive	New positive	New positive	Admission	New positive	New positive
Symptoms	+++	+	+	+	–	+	–	–	+	+	–
WBC (/mmc)	6430	9180	6280	10,250	4430	NA	8390	3440	7630	7150	3010
Lymphocytes (/mmc)	643	2552	1827	1158	1520	NA	3532	949	1381	2538	779.6
Neutrophils (/mmc)	5497	5333	3768	8887	2340	NA	4279	2208	5691	4082	1911.4
CRP (mg/dL)	5.7	1.3	0.3	0.3	0	NA	0.1	NA	7.7	0.1	0.3
PCT (ng/mL)	0.28	NA	0.13	<0.12	NA	NA	<0.12	NA	0.39	<0.12	NA
Creatinine (mg/dL)	0.7	0.5	0.9	0.66	0.98	NA	0.98	0.94	0.68	0.61	1.05
AST (UI/L)	76	26	24	17	37	NA	19	19	28	22	36
ALT (UI/L)	62	62	33	17	39	NA	18	12	47	25	21
CPK (UI/L)	618	28	92	65	NA	NA	38	NA	42	210	23
LDH (UI/L)	518	244	190	186	171	NA	165	154	188	237	154
d-Dimer (ng/mL)	NA	1350	340	558	NA	NA	242	NA	NA	166	NA
IgG anti-S1-S2 AU/ml	NA	64.5	102	23	52.9	32.3	NA	18.7	NA	170.0	199.0
Virus culture	NA	Negative	NA	NA	NA	Negative	Negative	Negative	NA	Negative	Negative

Abbreviations: *WBC* white blood cells, *CRP* C-reactive protein, *PCT* procalcitonin, *AST* aspartate-aminotransferase, *ALT* alanine aminotransferase, *CPK* creatine-phosphokinase, *LDH* lactate dehydrogenase, *NA* not available, +++ severe symptoms, + mild symptoms, − absence of symptoms

### Case 1

A 50-year-old man with a previous diagnosis of HBV infection developed fever on February 23, 2020, and cough on February 29, 2020. He was hospitalized on March 5, after being diagnosed with SARS-CoV-2 infection with a nasopharyngeal swab (Allplex™ 2019-nCoV Assay).

On admission, he presented a severe respiratory failure. Although antiviral and antimicrobial therapies were started, blood gas exchanges rapidly worsened, and he was intubated and transferred to the intensive care unit (ICU), where he remained for 33 days.

The clinical course in ICU was complicated with two episodes of ventilator-associated pneumonia (VAP) and a central venous catheter (CVC)–related bloodstream infection due to a carbapenem-resistant *Klebsiella pneumoniae*.

Two consecutive endotracheal samples resulted negative for SARS-CoV-2 on March 16 and March 19. He underwent further endotracheal samples (March 23, April 6, and April 8), two of which came indeterminate and the other two negatives (Allplex™ 2019-nCoV Assay).

**Fig. 1:**
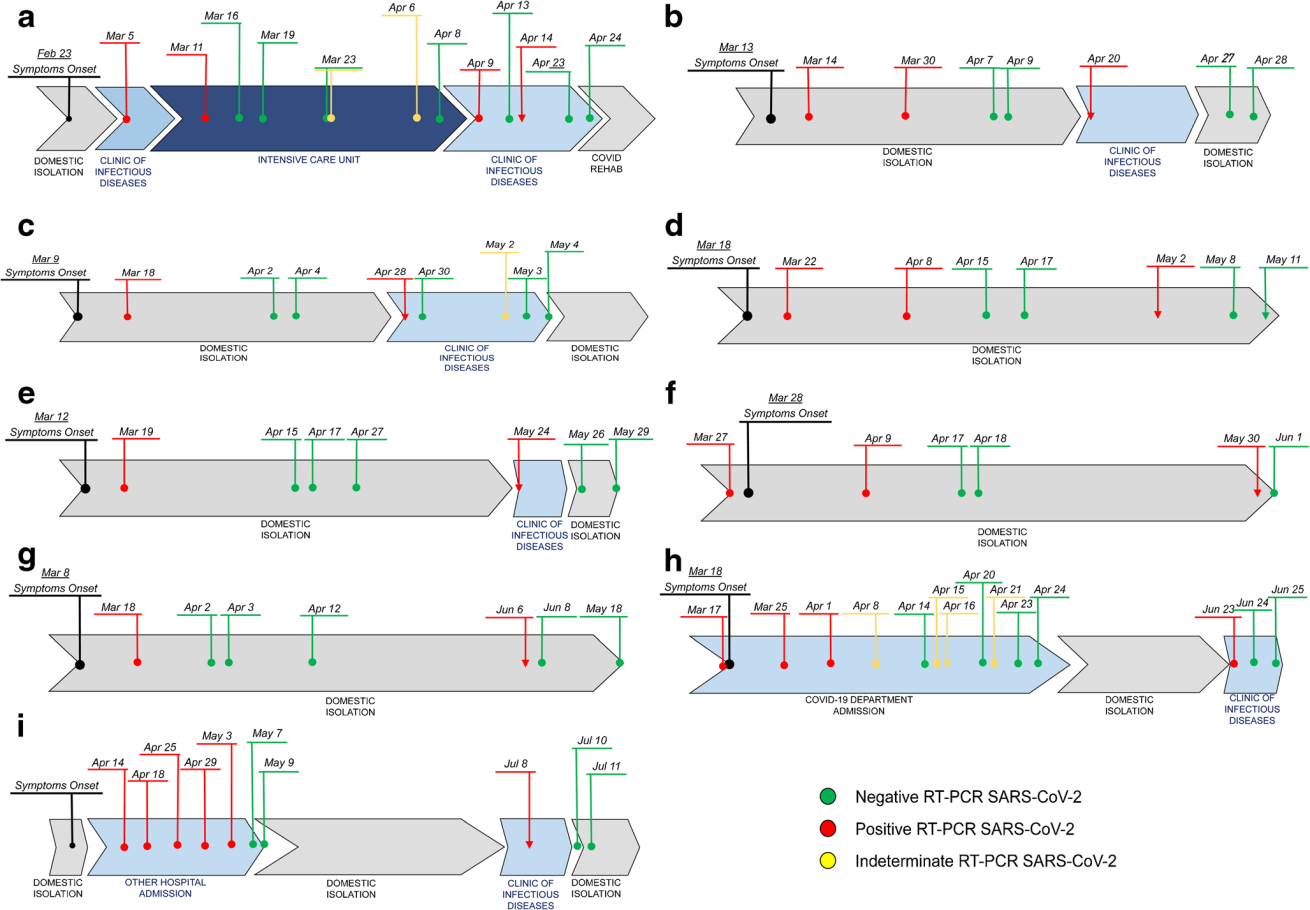
Respiratory sample timeline of each described case. Above the timeline: time of onset of symptoms and collection of samples. Under the timeline: movement of patient (e.g., hospitalization, discharge, domestic isolation). Black: onset of symptoms. Red: positive sample for severe acute respiratory syndrome (SARS-CoV-2). Green: negative sample for SARS-CoV-2. Yellow: indeterminate sample for SARS-CoV-2. Point: exam performed with traditional RT-PCR. Arrow: exam performed with Xpert® Xpress SARS-CoV-2, Cepheid

Between April 2 and April 8, qualitative serological tests (SCREEN® TEST COVID-19, ScreenItalia) resulted positive for both IgG and IgM. A nasopharyngeal swab on April 9 tested positive. Another sample collected on April 13 resulted negative while another one collected on April 14 was positive (nasopharyngeal swab; Xpert® Xpress SARS-CoV-2, Cepheid). On April 23 and 24, two additional nasopharyngeal swabs tested negative again (Fig. 1a).

His SARS-CoV-2 serology, collected on May 8, was compatible with a previous infection (IgG 64 AU/mL, LIASON® SARS-CoV-2 S1/S2 IgG, DIASORIN). As the clinical conditions were good, and the criteria for discharge were met, the patient was transferred to a COVID-dedicated rehabilitative ward.

The swab sample collected on April 14 (resulted positive) was stored, and culture-based virus isolation in Vero E6 cells was performed as previously described to evaluate the potential infectivity of the clinical specimen [6]. No virus was isolated, and viral RNA on supernatant was not detected (Xpert® Xpress SARS-CoV-2, Cepheid).

### Case 2

A 51-year-old man with a history of hypertension, tobacco use, and dyslipidemia developed fever until 38.3 °C with no other apparent symptoms on March 13, 2020. He underwent a nasopharyngeal swab which came positive for SARS-CoV-2 with RT-PCR (Allplex™ 2019-nCoV Assay). He was not hospitalized, as he did not complain of dyspnea and gradually became afebrile. He performed a follow-up nasopharyngeal swab, which came negative on April 7 (Allplex™ 2019-nCoV Assay). Afterwards, he went to the hospital for persistent retrosternal sense of weight with efforts. He was hospitalized once he underwent a screening swab test (Xpert® Xpress SARS-CoV-2, Cepheid) in the emergency room that tested positive on April 20. On April 21, his serology was compatible with a previous SARS-CoV-2 infection (IgG 23.6 AU/mL). He denied contact during quarantine with confirmed or possible COVID-19 cases.

In the following days, the pain disappeared, and his clinical features resulted normal. The patient was discharged in good clinical conditions with indication to repeat quarantine and swab tests that came negative for SARS-CoV-2 (Allplex™ 2019-nCoV Assay) on April 27 and 28 (Fig. 1b).

### Case 3

A 45-year-old woman with a history of iatrogenic hypothyroidism developed fever until 38 °C with arthro-myalgia, cough, headache, and conjunctival hyperemia from March 9, 2020. She also complained of dyspnea and desaturation from March 16. She was not in contact with a known case of SARS-CoV-2. She underwent a nasopharyngeal swab, which tested positive (Allplex™ 2019-nCoV Assay). She was not hospitalized. Fever and other symptoms disappeared after 2 weeks, while arthro-myalgia and headache persisted.

She performed two follow-up nasopharyngeal swabs on April 2 and 4, which tested negative (Allplex™ 2019-nCoV Assay) and a serologic test that was positive for IgG (SCREEN® TEST COVID-19, ScreenItalia).

While on quarantine, she referred a sense of chest weight and underwent computed tomography (CT) of the thorax that showed bilateral ground-glass opacities and interlobular septal thickening.

On April 28, she was hospitalized after a positive nasopharyngeal swab test; RNA gene “*N*” of SARS-CoV-2 was detected with a Ct value of 43 (Xpert® Xpress SARS-CoV-2, Cepheid). At the same time, her SARS-CoV-2 serology was positive for IgG 102 AU/mL.

During the hospitalization, pain progressively disappeared, and her clinical features resulted normal. No complications occurred. The patient was discharged in good clinical condition after two subsequent negative nasopharyngeal swabs (May 3 and 4, respectively; Allplex™ 2019-nCoV Assay) (Fig. 1c).

### Case 4

A 74-year-old man with no history of previous diseases developed fever until 38 °C with anosmia, dysgeusia, and lack of appetite on March 18, 2020. He denied dyspnea. His probable COVID-19 contact was his wife, so he underwent a nasopharyngeal swab, which came positive on March 22 (Allplex™ 2019-nCoV Assay). Considering his mild symptoms, he was not hospitalized.

He performed two follow-up pharyngeal swabs on April 15 and 17, which came negative (Allplex™ 2019-nCoV Assay). He also underwent a SARS-CoV-2 qualitative serologic test that was positive for IgG (SCREEN® TEST COVID-19, ScreenItalia).

During the following quarantine, he was always asymptomatic, but his CT of the chest showed bilateral interlobular septal thickening without interstitial pneumonia signs. Basing on this radiological sign, he underwent a nasopharyngeal swab test for SARS-CoV-2 that resulted positive on May 2 with detection of RNA genes “*E*” and “*N*” at Ct values of 32 and 35, respectively (Xpert® Xpress SARS-CoV-2, Cepheid).

The patient presented two subsequent negative nasopharyngeal swabs on May 8 and 11, both performed with RT-PCR assay and Xpert® Xpress SARS-CoV-2 (Cepheid) (Fig. 1d). At the same time, his SARS-CoV-2 serology was positive for IgG (52.9 AU/mL).

### Case 5

A 42-year-old woman with no history of previous diseases developed cough, mild dyspnea, sore throat, anosmia, and dysgeusia on March 12, 2020. She denied fever. She underwent a nasopharyngeal swab that tested positive on March 19 (Allplex™ 2019-nCoV Assay). Considering her mild symptoms, she was not hospitalized. During the quarantine period, she was always afebrile while the other symptoms gradually improved and completely disappeared on April 14.

She performed two follow-up nasopharyngeal swabs on April 15 and 17, which came negative (Allplex™ 2019-nCoV Assay). She also underwent a SARS-CoV-2 qualitative serologic test that was positive for IgG (SCREEN® TEST COVID-19, ScreenItalia).

On May 24, because of chest pain and arthro-myalgia, the patient was hospitalized. She underwent a nasopharyngeal swab test for SARS-CoV-2 that resulted positive with detection of genes “*E*” and “*N*” at Ct values of 44 and 37, respectively (Xpert® Xpress SARS-CoV-2, Cepheid). She was discharged 24 h later.

The patient presented two subsequent negative nasopharyngeal swabs on May 26 and 29, respectively (Allplex™ 2019-nCoV Assay) (Fig. 1e).

As in case 1, the swab sample of May 24 was cultured with Vero E6 cells. No viral replication was observed, and no viral RNA on the supernatant was detected.

### Case 6

A 77-year-old woman with chronic respiratory disease and cardiomyopathy had presumably a close contact with a positive case. She underwent nasopharyngeal swab on March 27 that came positive (Allplex™ 2019-nCoV Assay). The day after, she started complaining of generalized arthro-myopathy but no respiratory symptoms. She was considered healed when her nasopharyngeal swabs for SARS-CoV-2 came negative (May 17 and 19, Allplex™ 2019-nCoV Assay).

She tested positive in an ulterior test, performed on May 30 before a cardiological follow-up visit with detection of genes “*E*” and “*N*” at Ct values of 40 and 38, respectively (nasopharyngeal swab; Xpert® Xpress SARS-CoV-2, Cepheid). The following swab was performed on June 1 and came negative (RT-PCR assay) (Fig. 1f). At the same time, SARS-CoV-2 serology was compatible with a previous infection (IgG 32.3 AU/mL).

The sample of May 30 was inoculated onto Vero E6 cells, without any cytopathic effect development, and viral RNA on the supernatant was not found.

### Case 7

A 26-year-old physician working in a hospital in Northern Italy probably came in contact with a positive case during his professional activity. On March 8, 2020, he developed headache, arthro-myalgia, ageusia, anosmia, and dyspnea on exertion. He underwent a nasopharyngeal swab that tested positive on March 19 (RT-PCR assay). Considering his mild symptoms, he was not hospitalized. During the quarantine period, his clinical conditions were fine, but he kept on having mild cough and complaining of dyspnea.

He performed follow-up nasopharyngeal swabs on April 2, 3, and 12, which came negative (RT-PCR assay), and a high-resolution CT that showed two millimetric ground-glass opacities.

On May 5, 2020, he repeated the nasopharyngeal swab and serology test (method not available) that resulted negative.

On June 6, he was admitted to Perugia Hospital for a bone marrow donation and performed a new nasopharyngeal swab test for SARS-CoV-2 that resulted positive with detection of genes “*E*” and “*N*” at Ct values of 43 and 40, respectively (Xpert® Xpress SARS-CoV-2, Cepheid).

The patient presented two subsequent negative pharyngeal swabs on June 8 and 10, respectively (RT-PCR assay) (Fig. 1g). His SARS-CoV-2 serology was positive for IgG (18.7 AU/mL).

The swab sample of June 6, 2020, was inoculated in Vero E6 cell culture. No virus was isolated, and no viral RNA on the supernatant was detected.

### Case 8

A diabetic man, aged 50 years old, was hospitalized on March 18 for dyspnea after being diagnosed with COVID-19 with a nasopharyngeal swab (Allplex™ 2019-nCoV Assay). During the hospitalization, his respiratory exchanges gradually worsened, needing noninvasive ventilation. Tocilizumab, wide-range antibiotic treatment, hydroxychloroquine, and corticosteroids were also administered. His infection was classified as overcome once he collected two negative nasopharyngeal swabs (Allplex™ 2019-nCoV Assay) (Fig. 1h).

He underwent a follow-up CT scan that showed the ground-glass aspect of parenchyma and “crazy paving” phenomena in the inferior and superior right lobes. He was discharged on April 27.

The patient went to the hospital on June 23 for the persistence of headache, arthro-myalgias, asthenia, and insomnia. He was hospitalized again as a sample performed in the emergency room resulted positive (nasopharyngeal swab; Xpert® Xpress SARS-CoV-2, Cepheid). At the same time, SARS-CoV-2 serology showed IgG = 170 AU/mL. Subsequently, he was rapidly discharged after two negative pharyngeal swabs and negative SARS-CoV-2 culture in Vero E6 cells.

### Case 9

A 77-year-old man with a history of cardiomyopathy, atrial fibrillation, diabetes mellitus type 2, alcoholic cirrhosis, myelodysplastic syndrome, and hepatic neoformation developed fever with no other apparent symptoms on April 10, 2020. He was hospitalized after performing a nasopharyngeal swab with RT-PCR assay, which came positive (Allplex™ 2019-nCoV Assay). As he became afebrile and his parameters were in range, he was discharged after a follow-up pharyngeal swab that came negative on May 7 (Allplex™ 2019-nCoV Assay).

During quarantine, he was asymptomatic. On July 8, he went to the emergency room after seeing blood in his stools. He underwent a screening nasopharyngeal swab (Xpert® Xpress SARS-CoV-2, Cepheid) that tested positive with detection of genes “*E*” and “*N*” at Ct values of 33 and 35, respectively. His serology was compatible with a previous infection with SARS-CoV-2 (IgG = 199.0 AU/mL). He denied contact during quarantine with confirmed or possible COVID-19 cases.

The patient was discharged in good clinical conditions after two further swab tests for SARS-CoV-2 (Allplex™ 2019-nCoV Assay), which came negative on July 10 and 11 (Fig. 1i). The swab sample of June 6, 2020, was inoculated in Vero-E6 cell culture. No virus was isolated, and no viral RNA on the supernatant was detected.

### Systematic review results

A total of 82 papers matched the eligibility criteria [9–90]. No clinical or observational trials were found. Among those, there were 32 case reports and 50 case series. Furthermore, 5 review/minireview articles have been found [63, 64, 91–93]. Applying clinical criteria based on literature sources [94], we identified 1341 cases of COVID-19 that presented new positive respiratory samples after recovery. We analyzed data also including our nine presented cases, with a total of 1350. The characteristics of the patients are summarized in Table 2.

**Table 2:** Demographics, comorbidities, clinical presentation, and timing of respiratory samples for severe acute respiratory syndrome-coronavirus-2 (SARS-CoV-2) and outcome

	*N* 1350
Sex (*N* 1076), male percentage (%)	43.8
Age (*N* 160)	
Mean, years (range)	47.4 (4–91)
SD, years	19.3
Age (including case series, *N* 780)	
Weighted arithmetic mean, years	47.7
Hospital admission, days after symptom onset (*N* 81), mean (SD)	6.1 (7.6)
Charlson comorbidity index (*N* 107), median [IQR]	0 [0–2]
Presence of comorbidity (*N* 287), %	34.5
Symptoms at presentation	
Fever (*N* 320), %	66.3
Cough (*N* 317), %	52.3
Dyspnea (*N* 254), %	19.3
Nausea/diarrhea (*N* 276), %	8.3
Arthro-myalgia (*N* 281), %	12.8
Peripheral WBC (*N* 27)	
Mean, /mmc (range)	6281.5 (2900–13,400)
SD, /mmc	2858.0
Lymphocytes (*N* 33)	
Mean, /mmc (range)	1143.4 (60–2948)
SD, /mmc	636.4
Neutrophils (*N* 22)	
Mean, /mmc (range)	4740.4 (1595.9–9648.0)
SD, /mmc	2858.0
CRP (*N* 32)	
Mean, mg/dL (range)	2.8 (0.1–10.0)
SD, mg/dL	2.9
Pneumonia (*N* 316), %	82.0
ICU admission (*N* 232), %	2.6
Treatment (*N* 270), %	85.2
First positive respiratory samples	
Days from symptoms onset (*N* 120), mean (SD)	6.2 (4.7)
Fever (*N* 308), %	68.8
Symptoms (*N* 284), %	87.7
Type of samples (*N* 494)	
Swabs, %	98.8
Salivary tests, %	0.4
Sputum, %	0.8
Tracheal aspirate, %	0
First negative respiratory samples	
Days from symptoms onset (*N* 111), mean (SD)	19.1 (10.2)
Fever (*N* 1033), %	0.1
Symptoms (*N* 324), %	28.7
Type of samples (*N* 458)	
Swabs, %	98.3
Salivary tests, %	0.4
Sputum, %	1.1
Tracheal aspirate, %	0.2
New positive respiratory samples	
Days from symptoms onset (*N* 123), mean (SD)	34.5 (18.7)
Fever (*N* 805), %	5.7
Symptoms (*N* 1195), %	27.9
Type of samples (*N* 821)	
Swabs, %	97.7
Salivary tests, %	0.2
Sputum, %	1.9
Tracheal aspirate, %	0.1
SARS-CoV-2 IgG positivity (*N* 544), %	92.5
New negative respiratory samples	
Days from symptoms onset (*N* 76), mean (SD)	41.2 (21.5)
Fever (*N* 113), %	0
Symptoms (*N* 113), %	4.4
Type of samples (*N* 100)	
Swabs, %	95.1
Salivary tests, %	0.7
Sputum, %	4.3
Tracheal aspirate, %	0
Outcome (*N* 802)	
Recovery, %	91.6
Improvement, %	5.1
Still hospitalized, %	1.1
Death, %	2.1

Abbreviations: *N* number of patients, *STD* standard deviation, *IQR* interquartile range, *WBC* white blood cells, *CRP* C-reactive protein, *ICU* intensive care unit

Recurrence indistinctly occurred in the same rate in male and in female patients with a small predominance of female cases (male 43.8%, number of patients, *N* 1076). The patients were admitted to the hospital in a mean of 6.1 days (standard deviation, SD, 7.6 days; *N* 81) after symptoms onset. Only four patients were not hospitalized (cases 4, 5, 6, and 7). Recurrence was not associated with a specific age (mean age, 47.4 years old; SD, 19.3; range, 4–91 years old; *N* 160) or comorbidity (34.5% of patients had at least one comorbidity, *N* 287).

The most common symptoms at infection onset included fever (66.3%, *N* 320), cough (52.3%, *N* 317), dyspnea (19.3%, *N* 254), nausea or diarrhea (8.3%, *N* 276), and arthro-myalgia (12.8%, *N* 281).

As shown in Table 2, poor data are available about laboratory findings, but coherent to literature data concerning patients with SARS-CoV-2 infection [95]. Many cases also had CT scans or chest x-rays of patients which revealed the typical radiological sign of SARS-CoV-2 lung involvement (82.0%, *N* 316). Most patients underwent some antiviral treatment (85.2%, *N* 260).

Only six patients (included case 1 of our case series) required ICU admission (2.6%, *N* 232). In particular, case 1 and 3 other cases have been in ICU before the presumptive reactivation of SARS-CoV-2 infection, and only two patients needed ICU admission at the second episode [47, 55].

The first positive respiratory sample was collected in mean after 6.2 days (SD 4.7, *N* 120) from symptom onset. In that phase, 87.7% (*N* 284) of patients presented symptoms related to COVID-19, and 68.8% (*N* 308) of patients had fever.

In Table 2, we reported the first negative respiratory sample only if followed by a second negative sample. It occurred in mean after 19.1 days (SD 10.2 days, *N* 120). In this phase, 28.7% (*N* 324) of patients presented persisting but improving symptoms. Only in one case (0.1%, *N* 1033) did fever persist because of a *Klebsiella pneumoniae* bloodstream infection, documented at the same time.

These patients underwent follow-up respiratory sample collection after being declared recovered. New positive respiratory samples were found in mean 34.5 days (SD 18.7 days, *N* 123) after symptoms onset. Some patients presented fever (5.6%, *N* 805) and/or were still symptomatic (27.6%, *N* 1195). Most of them reported mild cough and respiratory symptoms. At that time, 92.5% of patients already had IgG against SARS-CoV-2.

Data concerning the following negative respiratory samples are available for 113 patients; none presented fever and 4% of the patients were mildly symptomatic.

The outcome was available for 802 patients. Among these, 91.6% recovered, 5.0% improved, 1.1% were still hospitalized at the time of data collection, and 2.1% died.

## Discussion

SARS-CoV-2 is a new coronavirus that since its discovery has been spreading all over the world, causing an impressive amount of deaths [96]. It is believed that patients infected with SARS-CoV-2 carry protective antibodies after recovery [97]. In literature, nonetheless, a series of cases of recurrences is reported, which means positivity for SARS-CoV-2 after two negative respiratory samples [98].

We need further data to determine risk factors, timing, and mechanisms that can cause it. Recurrence might probably be related to host factors (virologic status, underlying medical conditions, and therapy administered), characteristics of the virus itself, sample collection, and processing [98]. Currently, no reliable predictive marker of reactivation was found.

SARS-CoV-2 recurrence will be a vexing and persistent problem. Considering numerous patients infected or previously exposed to the virus, such a possibility poses a major public health burden [65].

The aim of the present study was to explore if the cases of suspected recurrence reported in the literature and in our clinic may be caused by a flawed sample or an incomplete clearance of the virus, or if effectively recovered people may be infected anew.

According to the presented data, the hypothesis of a recurrence seems to be improbable. Indeed, most of the patients were afebrile (5.7% of patients had fever), and symptoms were mild. These data depose for slow viral clearance and disease resolution instead of reactivation. Almost 97% of patients subsequently improved or recovered. Among 1350 cases, four patients died after presumptive recurrence, but it is not clear if the episode could be due to recurrence of COVID-19 [26]. Interestingly, Lafaye et al. described a geriatric case (case 1) with probable COVID-19 recurrence as he had clinical and radiological worsening, absence of neutralizing antibody, and positive cell culture during the second episode [77].

A real case of re-infection was described by Wang To et al. In this case, epidemiological, clinical, serological (IgG seroconversion), and genomic analyses confirmed that the patient had re-infection by a different strain of SARS-CoV-2 [74].

Osman et al. performed a review of re-positive cases after discharge from hospitals in China. They concluded that the re-positivization might be attributed to false-negative laboratory results and prolonged viral shedding, rather than re-infection [91]. However, the authors, in line with another review performed by Han et al., suggested that health authorities need to consider the importance of maintaining social distancing, even after the patients’ recovery [91, 92].

Cento et al. retrospectively analyzed data of 2521 recovered COVID-19. Negative-to-positive RT-PCR fluctuations occurred in 264/2521 patients, while none of them has ever shown a recurring of COVID-19 symptoms, regardless of RT-PCR results [82].

Kang et al. analyzed clinical and epidemiological information of 292 re-positive cases from the South Korea Centers for Disease Control and Prevention (KCDC). Patients were asymptomatic or complained about minor symptoms. The authors suggested that RNA of the “dead virus” remaining in the recovered patient’s body is amplified during the RT-PCR process. Furthermore, as SARS-CoV-2 does not cause chronic infection (seen as a latent stage), its reactivation is not virologically possible. They also concluded that comprehending the above evidence, re-positive cases are not contagious [42].

Concerning our nine cases, none presented severe disease or complications at the time of presumed recurrence. All tested patients (*N* 8) had antibody against the S1 and S2 subunit of SARS-CoV-2 spike protein with sufficient titer immediately before or during the presumptive recurrence. These data are in contrast with the hypothesis of recurrence of COVID-19.

Another important issue was to establish if these patients could be contagious again. To this purpose, 6 swab samples of the supposed “recurrence” in our patient cohort were stored, and the cultivability of SARS-CoV-2 in Vero E6 cells was assessed as previously described [6]. In all these cases, after 72 h of culture on Vero E6 cells, no virus replication has been observed, and viral RNA on supernatant was not detected. Since the first SARS-CoV-2 virus was isolated by our virology laboratory, many other swab samples have been cultured to isolate as much different virus strains as possible. We found that symptomatic patients with positive RT-PCR tests with Ct values < 25 easily provided cultivable SARS-CoV-2 viruses from their specimens. As previously reported, the results of RT-PCR from swab samples of re-positive patients of our cohort always showed Ct values above 24, so the uncultivability of these samples should be considered easily predictable [99]. This also strengthens the hypothesis of a “dead virus” that remained in the recovered respiratory tracts instead of a recurrence of infection. Furthermore, our results confirmed what previously declared by KCDC that tested negative viral cell culture of 108 re-positive cases [42].

Although these data should be confirmed in a larger number of cases, they strongly suggest that patients previously considered recovered and with a subsequent new sample positive for SARS-CoV-2 should not be considered contagious.

Limits of our study are the small number of cases; the incompleteness of some literature data, such as the detailed description of criteria for positivity and negativity; and the heterogeneity of the entire study cohort. Strengths are the analysis of all available literature about the recurrence of COVID-19 and the univocal answers to the issues.

## Conclusions

In conclusion, our results suggest that in most cases the presumptive recurrence is indeed a prolonged, not contagious, viral RNA persistence in the respiratory tract, probably due to a slow disease resolution. Further studies are necessary to definitively understand if a COVID-19 recurrence is possible and whether it could be considered as a real threat.

## Data Availability

Available on reasonable request.
